# Unveiling the Angiotensin-(1–7) Actions on the Urinary Bladder in Female Rats

**DOI:** 10.3389/fphys.2022.920636

**Published:** 2022-07-19

**Authors:** Gustavo B. Lamy, Eduardo M. Cafarchio, Bárbara do Vale, Bruno B. Antonio, Daniel P. Venancio, Janaina S. de Souza, Rui M. Maciel, Gisele Giannocco, Artur F. Silva Neto, Lila M. Oyama, Patrik Aronsson, Monica A. Sato

**Affiliations:** ^1^ Department Morphology and Physiology, Centro Universitario FMABC, Santo Andre, Brazil; ^2^ Department Biological Sciences, Federal University of Sao Paulo, Diadema, Brazil; ^3^ Department Medicine, Federal University of Sao Paulo, Sao Paulo, Brazil; ^4^ Department Physiology, Federal University of Sao Paulo, Sao Paulo, Brazil; ^5^ Department Pharmacology, Institute of Neuroscience and Physiology, Sahlgrenska Academy, University of Gothenburg, Gothenburg, Sweden

**Keywords:** intravesical pressure, ACE-2, Mas receptors, micturition, urinary bladder

## Abstract

Angiotensin-(1–7) is a peptide produced by different pathways, and regardless of the route, the angiotensin-converting enzyme 2 (ACE-2) is involved in one of the steps of its synthesis. Angiotensin-(1–7) binds to Mas receptors localized in different cells throughout the body. Whether angiotensin-(1–7) exerts any action in the urinary bladder (UB) is still unknown. We investigated the effects of intravenous and topical (*in situ*) administration of angiotensin-(1–7) on intravesical pressure (IP) and cardiovascular variables. In addition, the Mas receptors and ACE-2 gene and protein expression were analyzed in the UB. Adult female Wistar rats were anesthetized with 2% isoflurane in 100% O_2_ and submitted to the catheterization of the femoral artery and vein for mean arterial pressure (MAP) and heart rate (HR) recordings, and infusion of drugs, respectively. The renal blood flow was acquired using a Doppler flow probe placed around the left renal artery and the renal conductance (RC) was calculated as a ratio of Doppler shift (kHz) and MAP. The cannulation of the UB was performed for IP recording. We observed that angiotensin-(1–7) either administered intravenously [115.8 ± 28.6% angiotensin-(1–7) vs. −2.9 ± 1.3% saline] or topically [147.4 ± 18.9% angiotensin-(1–7) vs. 3.2 ± 2.8% saline] onto the UB evoked a significant (*p* < 0.05) increase in IP compared to saline and yielded no changes in MAP, HR, and RC. The marked response of angiotensin-(1–7) on the UB was also investigated using quantitative real-time polymerase chain reaction and western blotting assay, which demonstrated the mRNA and protein expression of Mas receptors in the bladder, respectively. ACE-2 mRNA and protein expression was also observed in the bladder. Therefore, the findings demonstrate that angiotensin-(1–7) acts in the UB to increase the IP and suggest that this peptide can be also locally synthesized in the UB.

## Introduction

Urinary bladder (UB) disorders affect the daily life of several patients worldwide due to mental and social discomfort (Kajiwara et al., 2004; Sureshkumar et al., 2009). It has been reported that UB symptoms show a higher prevalence in women (Aoki et al., 2017).

The storage and subsequent expulsion of urine from the bladder are mediated through both central and peripheral mechanisms that remain to be fully elucidated. Reflexes important for these functions are subject to direct cortical modulation, involving both facilitatory and inhibitory mechanisms. The Pontine Urine Storage Center, situated in the relative vicinity of the Pontine Micturition Center (PMC), which in turn initiates micturition (de Groat, 1998), regulates the storage of urine.

Neurons at several levels in the lumbosacral spinal cord and brain (raphe nucleus, reticular formation, locus coeruleus, red nucleus, PMC, hypothalamus, and cortex) have been shown to be connected to the UB, as demonstrated by retrograde labeling with pseudorabies virus (Nadelhaft et al., 1992).

Previous reports have shown that angiotensin II causes UB smooth muscle contraction in several species, including rabbits, monkeys, dogs, and humans (Falconieri-Erspamer et al., 1973; Lam et al., 2000). In the UB of adult humans, the action of angiotensin II is dependent on functional AT1 rather than on AT2 receptors (Andersson et al., 1992; Saito et al., 1993; Lam et al., 2000). Tanabe et al. (1993) have further demonstrated that AT1 receptors mediate contractions to angiotensin II in the rat bladder smooth muscle also *in vitro*. The UB dysfunction, which occurs after bladder outlet obstruction, has been attributed at least in part to angiotensin II acting on the AT1 receptor subtype (Yamada et al., 2009).

Angiotensin-(1–7) [Ang-(1–7)], an agonist on the Mas receptor, is synthesized in several pathways, for instance by cleavage of angiotensin I to angiotensin-(1–9) by ACE-2, and subsequent break-down by ACE and neutral endopeptidase 24.11 (NEP) (Chappell et al., 2000; Rice et al., 2004). In other words, angiotensin II cleavage into Ang-(1–7) by ACE-2 constitutes a physiologically important route (Vickers et al., 2002). Evidence in human ACE2 has demonstrated that its catalytic efficiency is 400-fold higher with angiotensin II as a substrate than with angiotensin I (Vickers et al., 2002; Rice et al., 2004). Likewise, this system (ACE-2/Ang-(1–7)/Mas receptor) is important in balancing detrimental actions of the ACE/Angiotensin II/AT-1 receptor axis, such as hypertension, atherosclerosis, and cardiac hypertrophy and fibrosis (Ferreira and Santos, 2005; Santos et al., 2018), albeit this mechanism alone does not explain all effects observed (Santos et al., 2000). Previous studies in Mas-knockout mice have demonstrated cardiovascular changes, ranging from induced hypertension and structural alterations in blood vessels to metabolic problems (Santos et al., 2006) (Xu et al., 2008), (de Moura et al., 2010) (Xu et al., 2008) (Santos et al., 2006).

Lamy et al. (2021) have demonstrated that Ang-(1–7) synthesized locally in the lateral preoptic area activates Mas receptors, which increases the IP without changes in renal conductance (RC) and cardiovascular variables. Despite Ang-(1–7) being found both peripherally and centrally (Santos and Campagnole-Santos, 1994), the role of Ang-(1–7) in the peripheral mechanisms involved in UB control is, to the best of our knowledge, largely unknown.

It is unclear whether peripheral Ang-(1–7) can act on the UB and, if so, would have any influence on UB dysfunctions or not. Furthermore, the presence of Mas receptors in the bladder or the existence of local synthesis of Ang-(1–7) mediated by ACE-2 in the UB is unknown. Thus, the present study focused on characterizing the effects of intravenous infusion and topical (*in situ*) administration of Ang-(1–7) on the UB and its impact on cardiovascular parameters. In addition, Mas receptor and ACE-2 gene and protein expression in the UB was investigated.

## Materials and Methods

### Animals

Female Wistar rats (∼230–250 g, 14–16 weeks old) supplied by the Animal Facility of the Faculdade de Medicina do ABC/Centro Universitario FMABC were used. The animals were housed in plastic cages in groups of four rats each and had free access to standard chow pellets (Nuvilab®) and tap water *ad libitum*. The animals were in an air-conditioned room with a temperature between 20℃ and 24℃ and a 12:12-h light–dark cycle. The humidity in the animal room was controlled and maintained at ∼70%. All procedures of this study were carried out in accordance with the National Institutes of Health Guide for the Care and Use of Laboratory Animals. This study was approved by the Animal Ethics Committee of the Faculdade de Medicina do ABC/Centro Universitario ABC (protocol number 13/2017).

### Cannulation of the Urinary Bladder

A small incision was carried out in the apex of the UB wall for insertion of a polyethylene tubing (PE-50 connected to PE-10, Clay Adams, NJ, United States) filled with saline as previously described by Cafarchio et al. (2016, 2018, and 2020), Magaldi et al. (2020), and Lamy et al. (2021). Tissue glue was used to keep the catheter fixed on the bladder wall for intravesical pressure (IP) recordings in a data acquisition system (PowerLab 16 SP, AD Instruments, Castle Hill, AU). The urethra outlet was not ligated to allow the bladder voiding if necessary. The baseline IP value was set at ∼6–8 mmHg, and the adjustment of the baseline IP was done by infusion of saline or urine withdrawal through the catheter inserted into the bladder. Percent changes in IP (%ΔIP) were calculated as [(peak IP response—baseline IP)/baseline IP] × 100.

### Cannulation of the Femoral Artery and Vein

The catheterization of the femoral artery and vein was performed through the insertion of a polyethylene tubing (PE-50 connected to PE-10, Clay Adams, NJ, United States) to measure pulsatile arterial pressure (PAP), mean arterial pressure (MAP), and heart rate (HR) in the data acquisition system (PowerLab 16 SP, AD Instruments, Castle Hill, AU) and injections of drugs, respectively.

### Measurement of Regional Blood Flow

Rats underwent a midline laparotomy for placement of a miniaturized pulsed Doppler flow probe (0.8 mm in diameter, Iowa Doppler Products, Iowa City, IA, United States) around the left renal artery for indirect measurement of the blood flow. The probe was connected to a Doppler flowmeter (Department of Bioengineering, The University of Iowa, Iowa City, IA, United States), and the amplified signal was digitalized in a data acquisition system (PowerLab 16 SP, AD Instruments, Castle Hill, AU). Details about the readability of this method for estimation of the blood velocity and regarding the Doppler technique have been previously described by Haywood et al. (1981). Relative renal vascular conductance was calculated as the ratio of Doppler shift (kHz) and MAP (mmHg). Results were presented as percent change from the baseline [(final conductance − initial conductance)/initial conductance × 100].

### Topical (*in situ*) Drug Administration

The topical administration of Ang-(1–7) was always performed at a dose of 100 ng/ml in a volume of 0.1 ml directly onto the surface of the UB.

### Intravenous Drug Administration

Ang-(1–7) was administered intravenously at a dose of 0.24 μg/kg/min using an infusion pump (Insight Ltda, Ribeirão Preto, SP). The solution of Ang-(1–7) was prepared at a concentration of 0.24 μg/ml.

### Experimental Protocols

#### Evaluation of Intravenous Administration of Angiotensin-(1–7) on Intravesical Pressure and Cardiovascular Variables in Female Wistar Rats (*N* = 6)

**FIGURE 1:**
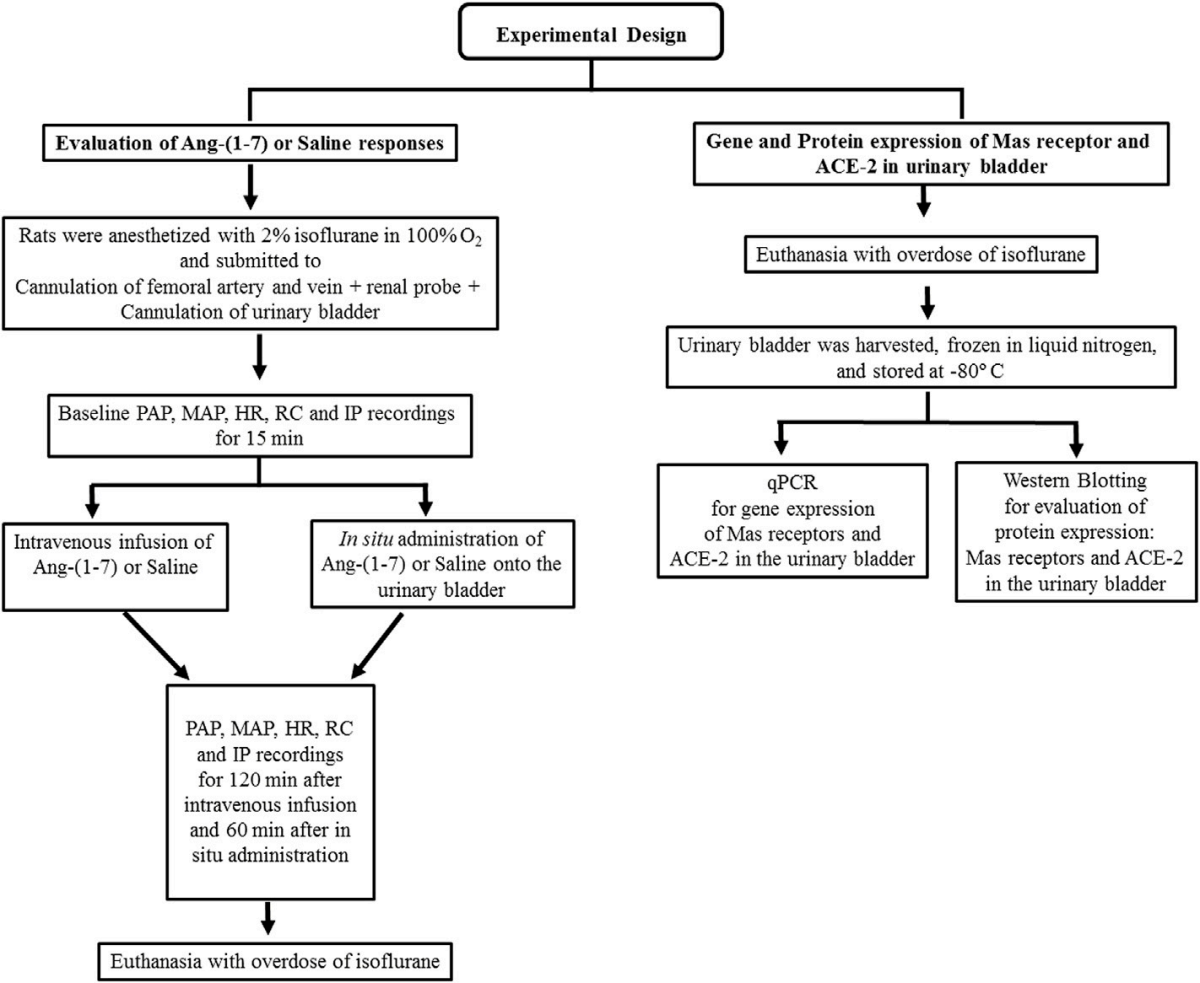
Flowchart showing the experimental design of the study.

Rats anesthetized with isoflurane 2% in 100% O_2_ were submitted to cannulation of the femoral artery and vein. A laparotomy was performed for placement of a miniaturized Doppler flowmeter probe around the left renal artery to measure the renal blood flow. The UB was also cannulated with a polyethylene tubing for IP recording. After baseline measurement of IP, arterial pressure, HR, and renal blood flow for 15 min, an infusion of Ang-(1–7) 0.24 μg/kg/min or saline (vehicle) was performed intravenously and the variables were measured for another 120 min (Figure 1). We have chosen the dose of Ang-(1–7) 0.24 μg/kg i.v. because it is equivalent to the lowest dose used by Durik et al. (2012) with stabilized Ang-(1–7) analog molecule that did not cause changes in cardiac parameters.

#### Evaluation of Topic (*in situ*) Administration of Angiotensin-(1–7) on Intravesical Pressure and Cardiovascular Variables in Female Wistar Rats (N = 6)

The animals were anesthetized with isoflurane 2% in O_2_ 100% and submitted to the same surgical procedures described in section 7.1. After baseline IP, arterial pressure, HR, and renal blood flow recordings for 15 min, topical (*in situ*) administration of Ang-(1–7) was done at a dose of 100 ng/ml in a volume of 0.1 ml or saline (0.1 ml). The topical administration was performed by dropping Ang-(1–7) or saline onto the surface of the UB, and the variables were measured for another 60 min (Figure 1).

This protocol was carried out to verify if Ang-(1–7) would elicit direct effects on the detrusor muscle of Wistar rats.

#### Gene Expression of Mas Receptors and ACE-2 in the Urinary Bladder (*n* = 6)

As shown in Figure 1, animals used in this protocol were not previously instrumented for IP and cardiovascular recordings. Rats were deeply anesthetized with isoflurane 4% in 100 O_2_ and submitted to a laparotomy for UB withdrawal. The bladder was immediately frozen in liquid nitrogen and stored at −80°C in an ultrafreezer until the day of total RNA extraction with the TRizol® reagent. The procedures for Mas receptors, ACE-2, and cyclophilin A gene expression were carried out through quantitative real-time polymerase chain reaction (qPCR) as follows.

Isolation of total mRNA from frozen UB samples was made with TRIzol Reagent® (Thermo Fisher Scientific) as suggested by the manufacturer’s protocol. mRNA integrity was analyzed via agarose gel electrophoresis, and mRNA purity reached the following criteria: A260/280 ≥ 1.8. The extracted total mRNA concentration was measured using a NanoDrop^TM^ (One-One c) spectrophotometer (Thermo Fisher Scientific). We used 1 μg of total mRNA for the reverse transcription reaction. Complementary DNA (cDNA) synthesis was obtained using the ImProm-II^TM^ Reverse Transcription System (Promega, Madison, WC, United States), following the manufacturer’s protocol. qPCR was performed using 2 μl of cDNA and the Eva Green^TM^ qPCR Mix Plus (Solis BioDyne, Tartu, Estonia) in the ABI Prism 7500 Sequence Detection System (Applied Biosystems, Foster City, CA) to amplify specific primers sequences for the Mas receptors, ACE-2, and cyclophilin A. The forward and reverse primers sequences (Thermo Fisher Scientific) for rats are shown below:

Mas receptor:

(forward) 5′- CCT​GCA​TAC​TGG​GAA​GAC​CA-3′

(reverse) 5′- TCC​CTT​CCT​GTT​TCT​CAT​GG-3′

ACE-2:

(forward) 5′- TTG​AAC​CAG​GAT​TGG​ACG​AAA-3′

(reverse) 5′- GCC​CAG​AGC​CTA​CGA​TTG​TAG​T-3′

Cyclophilin A:

(forward) - 5′-CCC​ACC​GTG​TTC​TTC​GAC​AT-3′

(reverse) - 5′-CTG​TCT​TTG​GAA​CTT​TGT​CTG​CAA-3′

The internal control (housekeeping gene) used in this protocol was cyclophilin A. The procedure followed an initial step of 10 min at 95°C, followed by 45 cycles of 20 s each at 95°C, 20 s at 58°C, and 20 s at 72°C. Gene expression was determined using threshold cycle (CT) (arbitrary units, A.U.), and all values were expressed using cyclophilin A as an internal control.

#### Protein Expression of theas Receptor and ACE-2 in the Urinary Bladder (*n* = 6)

As described in Figure 1, we used different groups of rats for protein and gene expression. Animals were deeply anesthetized with isoflurane 4% in 100% O_2_. A laparotomy was carried out, and the UB was harvested and immediately frozen in liquid nitrogen. Bladder samples were stored at −80°C in an ultrafreezer for later determination of Mas receptor and ACE-2 protein expression in the UB via western blotting. The rats used in this protocol were not previously submitted to any surgery or IP and cardiovascular recordings. The procedures for the western blotting assay followed the steps described below.

The lysate of UB samples was obtained using radioimmunoprecipitation assay lysis and extraction buffer, added with a mixture of protease and phosphatase inhibitors (Thermo Fisher Scientific). The samples were homogenized in lysis buffer, incubated on ice for 10 min, and centrifuged at 7000 g for 5 min at 4°C. The supernatant containing the soluble proteins was stored at −80°C. The protein concentration was determined using NanoDrop^TM^ (One-One c) spectrophotometer (Thermo Fisher Scientific). The total proteins were separated on a 10% sodium dodecyl sulfate–acrylamide gel and transferred to the nitrocellulose membrane (Bio-rad) by electrophoresis using the Trans-blot Turbo Transfer device (Bio-rad). Ponceau solution was used to stain the membrane to check successful transfer. The membrane was photographed in the Chemidoc device (Bio-rad) for the determination of total protein by densitometry using the Image Lab^TM^ software (Bio-rad). Afterward, the membrane was washed with milli-Q water at least three times. The membrane was incubated for 1 h with 5% nonfat milk in Tris-buffered saline- 0.1% Tween 20 (TBS-T). Then, the solution was discarded and the membrane was incubated at 4°C, overnight, with a polyclonal primary antibody specific for the Mas receptor (rabbit anti-Mas, Novus Biologicals, catalog # NBP1-78444) and for ACE-2 (rabbit anti-ACE-2, Cloud-Clone Corp., catalog # PAB886Ra01) diluted to a concentration of 1:250 in TBS-T. The blots were washed with TBS-T and afterward, incubated with goat–anti-rabbit secondary antibody (Alexa Fluor 488, Thermo Fischer Scientific) in a 1:10,000 dilution for 1 h, which causes a chemiluminescent reaction. The membrane was filmed in the Chemidoc device (Bio-rad). The Image Lab software ^TM^ (Bio-rad) was used to quantify the densitometry of the blot containing the corresponding protein of interest. The optical density (OD) of Mas receptors and ACE-2 was normalized by the expression of total protein, as described in a previous study by Lamy et al. (2021).

### Statistics

The Kolmogorov–Smirnov test was carried out to evaluate the normality of data distribution. Results were expressed as mean ± S.E.M as they showed a normal distribution. Unpaired Student’s t-test was used for comparison between the %ΔIP or %ΔRC responses evoked by Ang-(1–7) versus saline administered intravenously or *in situ* onto the UB. Comparisons of MAP and HR before and after i.v. or *in situ* Ang-(1–7) or saline were analyzed using paired Student´s t-test. The statistical software package Sigma Stat 3.5 was used for all the analyses, and the data were considered significant at *p* < 0.05.

## Results

### Responses in Intravesical Pressure and Cardiovascular Parameters Evoked by Intravenous Administration of Angiotensin-(1–7) in Wistar Rats (*n* = 6).

**FIGURE 2:**
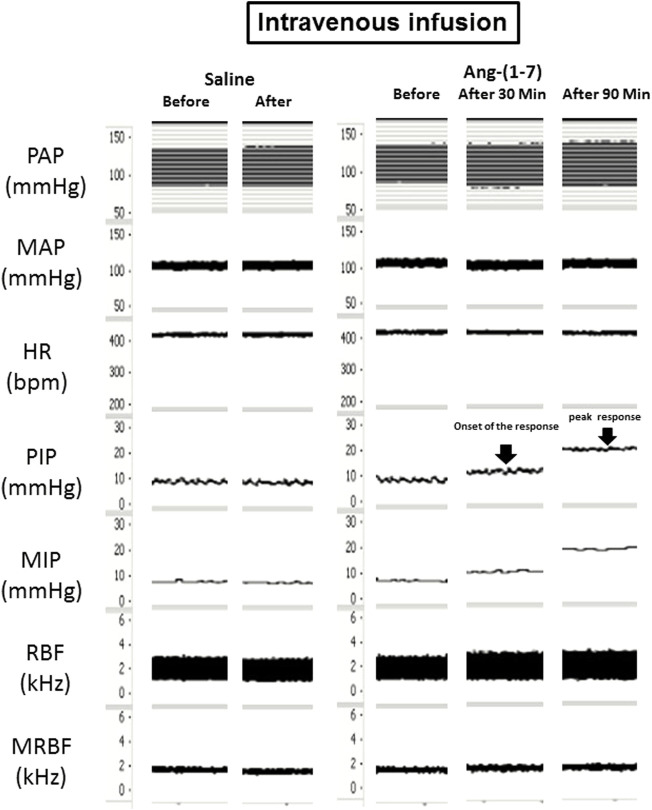
Tracings showing the baseline pulsatile arterial pressure (PAP, mmHg), mean arterial pressure (MAP, mmHg), heart rate (HR, bpm), pulsatile intravesical pressure (PIP, mmHg), mean intravesical pressure (MIP, mmHg), renal blood flow (RBF, kHz) and mean renal blood flow (MRBF, kHz) and the responses induced by intravenous administration of angiotensin-(1–7) (0.24 μg/kg) or saline.

**FIGURE 3:**
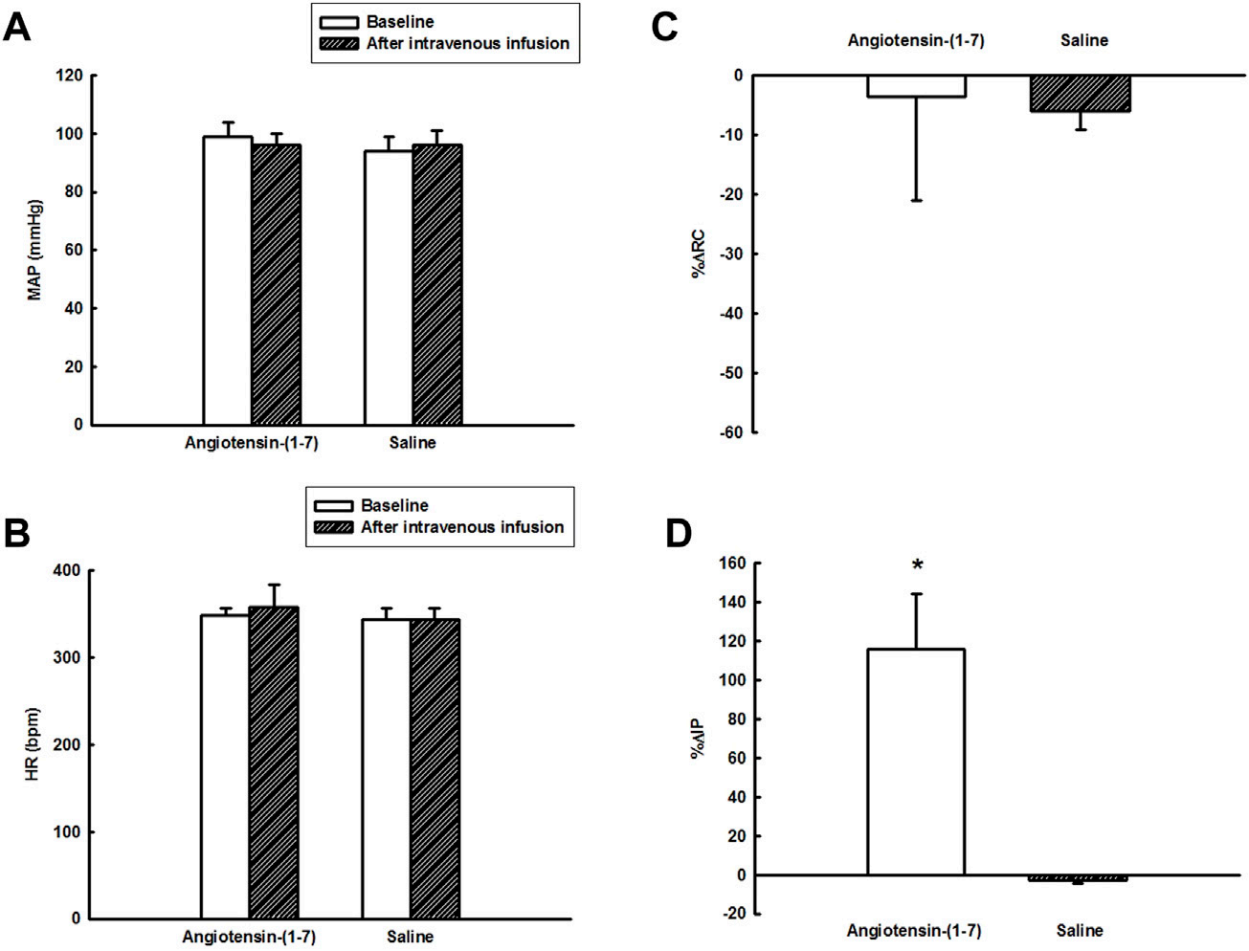
**(A)** Mean arterial pressure (mmHg) and **(B)** heart rate (bpm) at baseline and after intravenous administration of angiotensin-(1–7) (0.24 μg/kg) or saline, **(C)** percent change in renal conductance (%ΔRC), and **(D)** percent change in intravesical pressure (%ΔIP) evoked by intravenous administration of angiotensin-(1–7) (0.24 μg/kg) or saline (*n* = 6). *p < 0.05 vs. saline (Student´s t-test).

Before infusions of saline or Ang-(1–7) intravenously, the baseline MAP was 94 ± 5 mmHg (before saline) and 99 ± 5 mmHg [before Ang-(1–7)], the HR was 344 ± 13 bpm (before saline) and 348 ± 9 bpm [before Ang-(1–7)], and the IP was 8.38 ± 0.64 mmHg (before saline) and 7.72 ± 0.81 mmHg [before Ang-(1–7)] (*n* = 6) (Figures 2, 3A,B).

The intravenous administration of Ang-(1–7) significantly increased IP (115.8 ± 28.6%, *p* < 0.05) compared to saline (−2.9 ± 1.3%). The onset of IP increase induced by Ang-(1–7) administered intravenously had a latency of ∼30 min and the peak response was achieved at ∼90 min after the injection (Figures 2, 3D). After 120 min, the IP values recovered to the baseline values.

However, no significant changes were observed in MAP [2 ± 1 mmHg after saline vs. −3 ± 3 mmHg after Ang-(1–7)], HR [1 ± 2 bpm after saline vs. 10 ± 18 bpm after Ang-(1–7)], and RC [6.0 ± 3.2% after saline vs. −3.6 ± 17.4% after Ang-(1–7)] after intravenous infusion of saline or Ang-(1–7) (Figures 2, 3A–C).

### Responses in Intravesical Pressure and Cardiovascular Parameters Elicited by Topic (*in situ*) Administration of Angiotensin-(1–7) Onto the Urinary Bladder in Wistar Rats (N = 6).

**FIGURE 4:**
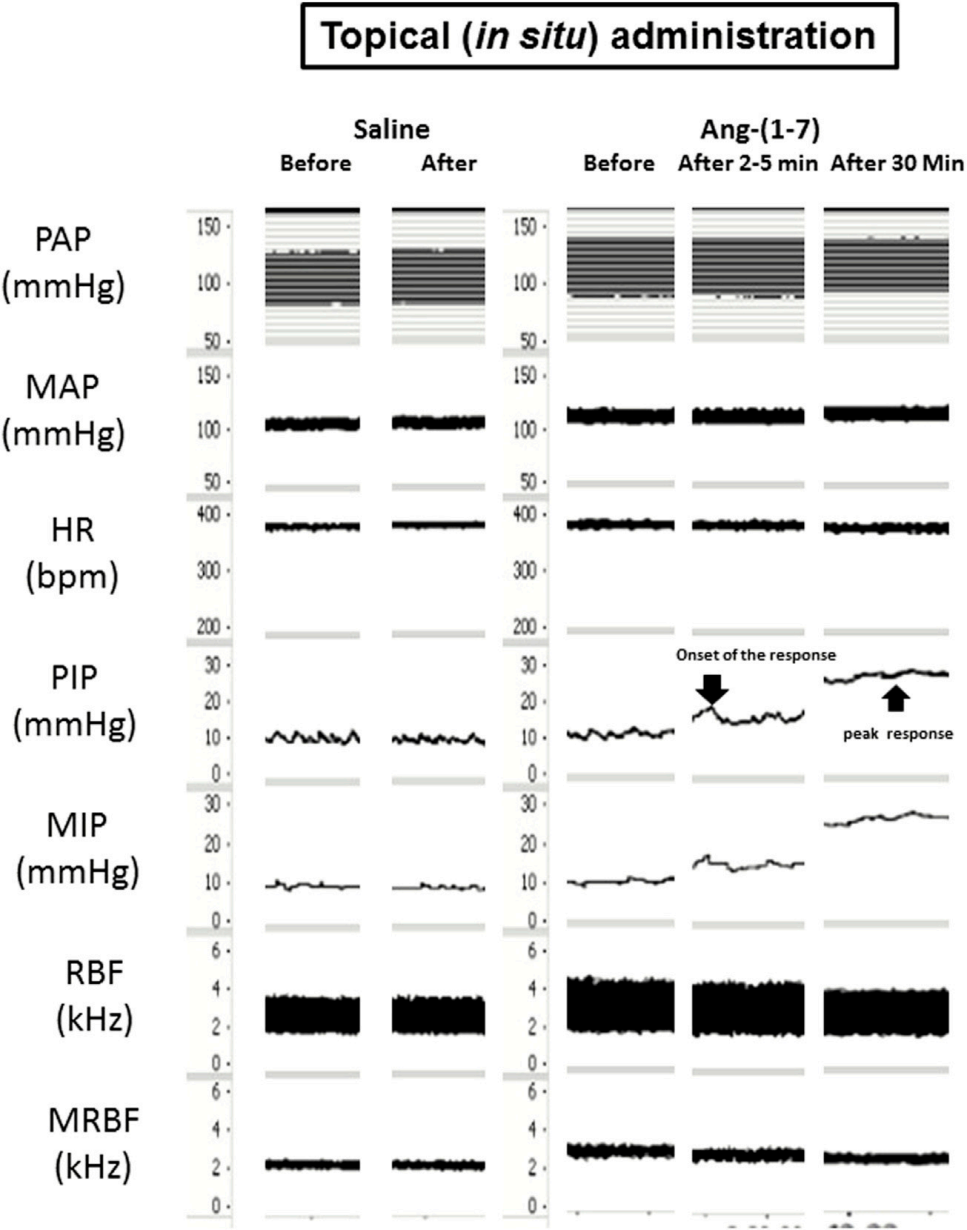
Tracings showing the baseline pulsatile arterial pressure (PAP, mmHg), mean arterial pressure (MAP, mmHg), heart rate (HR, bpm), pulsatile intravesical pressure (PIP, mmHg), mean intravesical pressure (MIP, mmHg), renal blood flow (RBF, kHz) and mean renal blood flow (MRBF, kHz) and the responses yielded by *in situ* administration of angiotensin-(1–7) (100 ng/ml, 0.1 ml per rat) or saline (0.1 ml) onto the urinary bladder.

**FIGURE 5:**
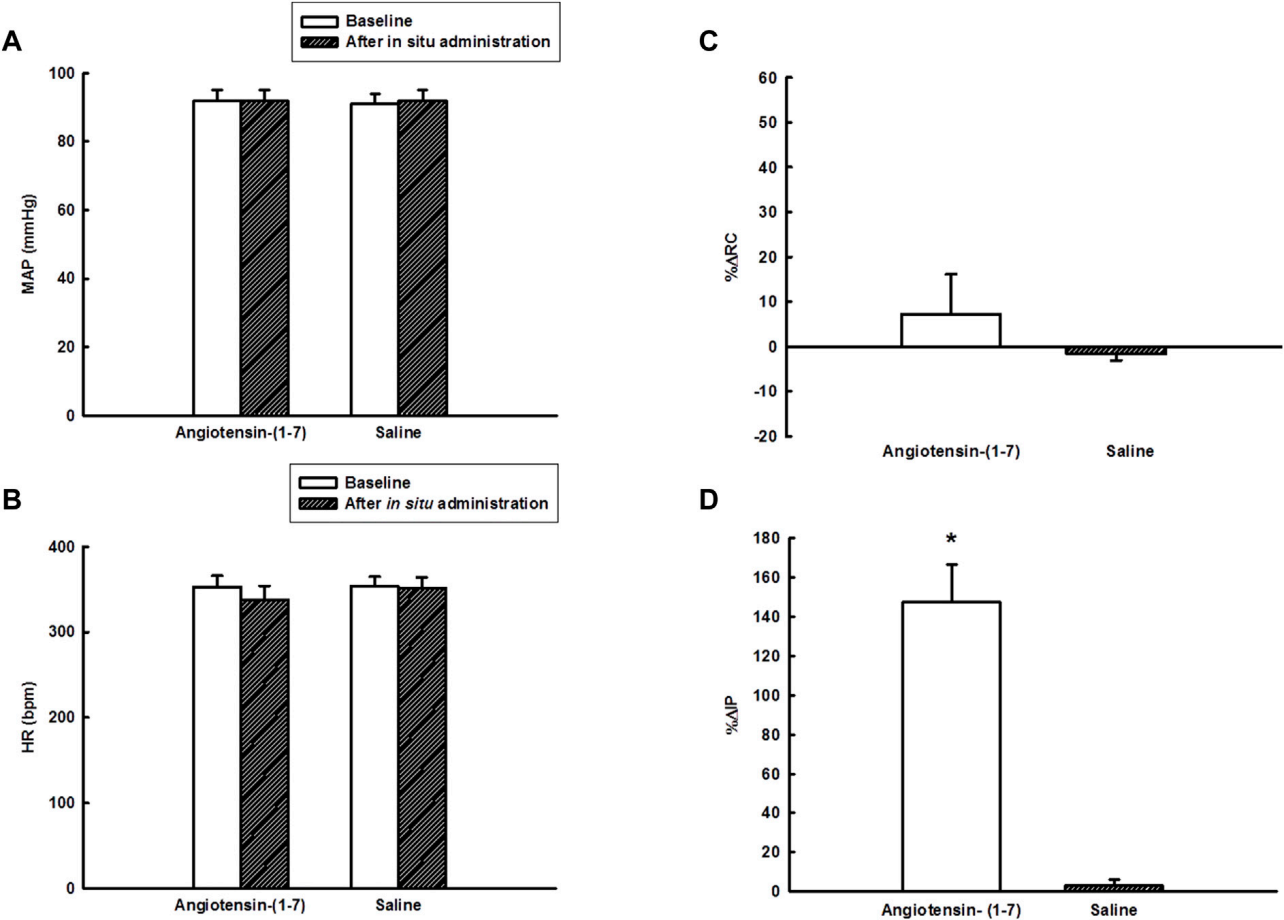
**(A)** Mean arterial pressure (mmHg) and **(B)** heart rate (bpm) at baseline and after *in situ* administration of angiotensin-(1–7) (100 ng/ml, 0.1 ml per rat) or saline (0.1 ml), **(C)** percent change in renal conductance (%ΔRC), and **(D)** percent change in intravesical pressure (%ΔIP) elicited by *in situ* administration of angiotensin-(1–7) (100 ng/ml, 0.1 ml per rat) or saline (0.1 ml) on the urinary bladder (*n* = 6). *p < 0.05 vs. saline (Student´s t-test).

Before application of saline or Ang-(1–7) *in situ* on the UB, the baseline MAP was 91 ± 3 mmHg (before saline) and 92 ± 3 mmHg [before Ang-(1–7)], the HR was 354 ± 11 bpm (before saline) and 353 ± 13 bpm [before Ang-(1–7)], and the IP was 5.98 ± 0.65 mmHg (before saline) and 6.85 ± 0.64 mmHg [before Ang-(1–7)] (*n* = 6) (Figures 4, 5A,B).

The topic (*in situ*) administration of Ang-(1–7) onto the UB significantly increased the IP (147.4 ± 18.9%, *p* < 0.05) compared to saline (3.2 ± 2.8%). The onset of IP increase evoked by Ang-(1–7) administered *in situ* onto the UB had a latency of ∼2–5 min and the peak response was observed at ∼30 min after the administration (Figures 4, 5D). After 60 min, the IP values drop back to baseline values.

Nevertheless, the topic (*in situ*) administration of Ang-(1–7) on the UB elicited no change in MAP (0.3 ± 1 mmHg vs. 0.5 ± 0.6 mmHg, saline), HR (−15 ± 4 bpm vs. −2 ± 1 bpm, saline) and RC (7.2 ± 8.9% vs. −1.6 ± 1.5%, saline) compared to saline administration (Figure 4, 5A–C).

### Determination of Mas Receptors and ACE-2 Gene Expression in the Urinary Bladder (N = 6)

**FIGURE 6:**
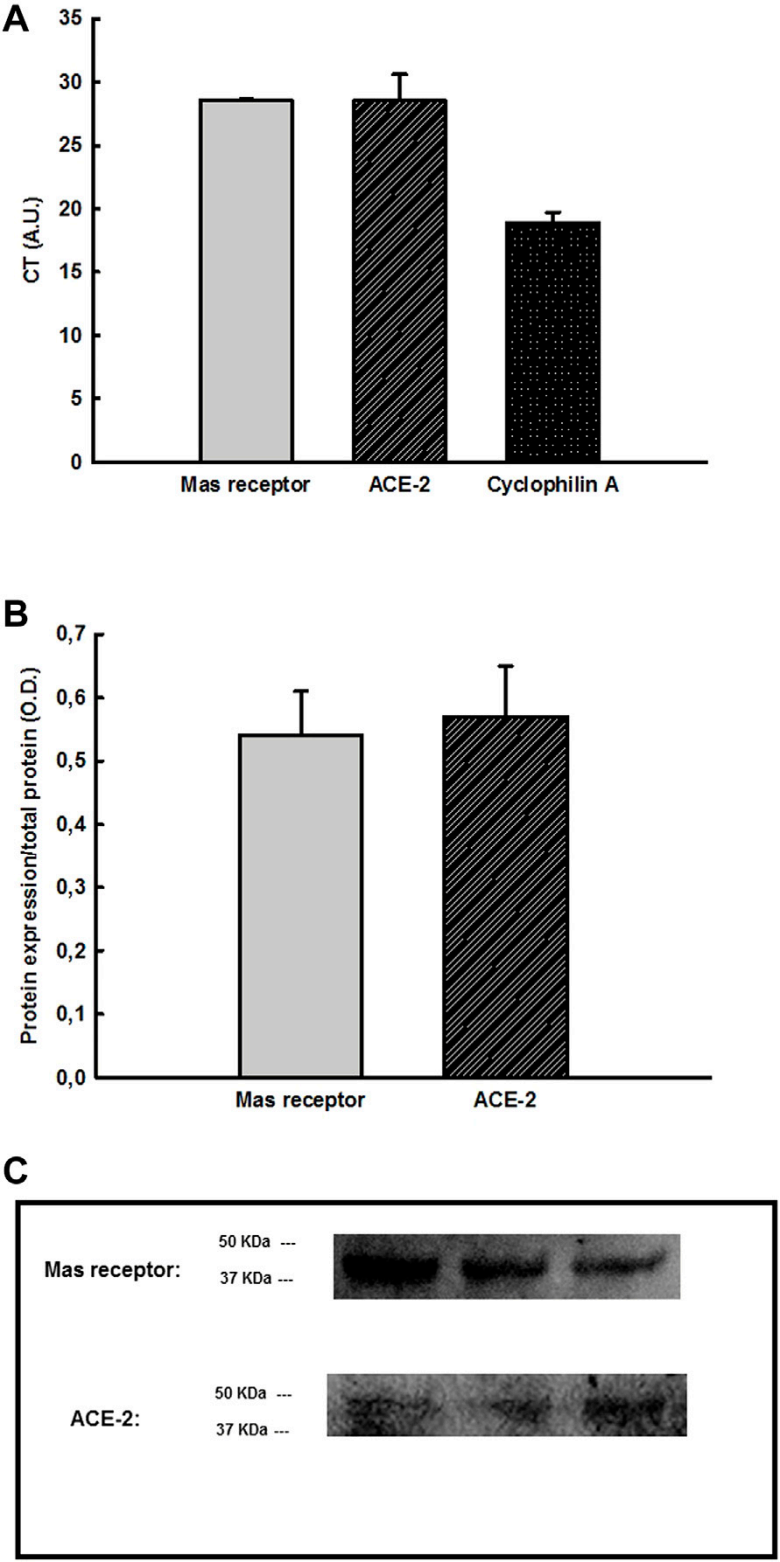
**(A)** CT values obtained by qPCR showing the Mas receptors, ACE-2, and cyclophilin A (housekeeping gene) gene expression in the urinary bladder (UB) (*n* = 6), **(B)** Mas receptors and ACE-2 protein expression normalized by total protein in the UB by western blotting assay (*n* = 6), **(C)** Blots labeled for Mas receptors and ACE-2 in the UB samples by western blotting. Abbreviation: OD, Optical density.

We observed that the Mas receptors (CT = 28.56 ± 0.124 arbitrary units, A.U.) and ACE-2 gene (CT = 28.54 ± 2.05 A.U.), as well as the housekeeping gene cyclophilin A (18.89 ± 0.81 A.U.), were expressed, and thereby, these genes are present in the UB samples (N = 6) (Figure 6A).

### Determination of Protein Expression of Mas Receptors and ACE-2 in the Urinary Bladder (N = 6)

We found by western blotting assay that Mas receptors (0.54 ± 0.07 O.D.) and ACE-2 (0.57 ± 0.08 O.D.) proteins are expressed in the UB samples (N = 6) (Figures 6B,C).

## Discussion

Our data demonstrated that intravenous infusions of Ang-(1–7) elicited a marked increase in IP compared to saline. Nevertheless, the RC, arterial pressure, and HR were not affected by i.v. Ang-(1–7) in the dose used in this study. Those findings indicate that the increase in IP is not likely dependent on higher urinary volume caused by an increase in the glomerular filtration rate. The topical (*in situ*) administration of Ang-(1–7) also yielded an increase in IP and elicited no changes in cardiovascular parameters in the rats, showing similar effects compared to intravenously infused Ang-(1–7). It is noteworthy that the latency for the increase in intravesical evoked by *in situ* application of Ang-(1–7) on the UB was shorter (5 min) compared to the response elicited by intravenous administration (30 min). We also demonstrated the presence of Mas receptors in the UB either by gene or protein expression, which suggests that Ang-(1–7) is likely binding to these receptors for increasing the IP. Furthermore, we showed the existence of gene and protein expression of ACE-2 in the UB samples, which is suggestive that Ang-(1–7) can be locally produced in the UB cells.

The regulation of UB function is dependent on the autonomic and somatic nervous systems. Cholinergic (via muscarinic receptors) and adrenergic (via α and β receptors) transmissions, as well as noncholinergic/nonadrenergic mechanisms (NCNA), exert an important function in the storage of urine and voiding (Juszczak and Maciukiewicz, 2017). Several neurotransmitters of the NCNA branch of the autonomic nervous system, for instance, ATP, substance P, and neuropeptide Y, can play stimulatory or inhibitory neuromodulation of cholinergic, adrenergic, or purinergic transmission in the lower urinary tract (Hoyle, 1994; Juszczak and Maciukiewicz, 2017). *In vitro* studies performed by Tanabe et al. (1993) demonstrated that the AT1 receptors, instead of AT2 receptors, mediate the contractions elicited by angiotensin II in the smooth muscle of rat UB strips. The function of the renin–angiotensin–aldosterone system on the lower urinary tract is not fully understood, and our study is the first to show the effects of intravenous Ang-(1–7) or *in situ* Ang-(1–7) onto the UB on the IP, as well the existence of Mas receptors and ACE-2 in the bladder by gene and protein expression.

Although Ang-(1–7) promoted increases in IP after *in situ* administration onto the UB, it is not possible to infer physiologically whether the plasma Ang-(1–7) or this peptide locally synthesized in the cells of the UB yields the increases in IP. We have shown the existence of gene and protein expression of ACE-2, which is the enzyme responsible for the synthesis of Ang-(1–7) breaking the angiotensin I (Chappell et al., 2000; Rice et al., 2004) or alternatively from the cleavage of angiotensin II (Vickers et al., 2002). This allows us to hypothesize that Ang-(1–7) is locally produced in the cells of the UB and possibly exerts a paracrine or autocrine action and thereby could increase the IP.

Previous studies have demonstrated that intrarenal infusion of Ang-(1–7) at a rate of 0.1 and 1 nmol.min^−l^.kg^−l^ had minimal effects on renal blood flow and arterial pressure but raised the urinary excretion of sodium and water in comparison to the control (saline-infused) group (Handa et al., 1996). This effect was attributed to a possible action of Ang-(1–7) in the proximal tubule cells, where Ang-(1–7) could inhibit an ouabain-sensitive Na^+^-K^+^-ATPase. This inhibitory action of Ang-(1–7) was attributed mainly to a non-AT1, non-AT2 angiotensin receptor, and in minor proportion to AT-1 receptors (Handa et al., 1996). Nevertheless, the study of Handa et al. (1996) has not investigated whether the Mas receptor could be involved or not in the response evoked by Ang-(1–7). Despite we have demonstrated that Ang-(1–7) administered either intravenously or *in situ* has not affected the renal blood flow, we have not evaluated the urine volume compared to saline administration (control group), which is a limitation of this study. Considering that the topical (*in situ*) administration of Ang-(1–7) onto the UB elicited an increase in IP with a latency of 2–5 min, we do not believe that this response would be dependent on an increase in the urinary volume. By contrast, we cannot assure that the marked increase in IP yielded by intravenous infusion of Ang-(1–7) could cause an increase in UB dependent on an intrarenal action due to the long latency for the onset of the response of roughly 30 min.

On the other hand, another hypothesis is that Ang-(1–7) infused intravenously could also act centrally in the lateral preoptic area and bind to the Mas receptors of this area. Previous report has shown that Ang-(1–7) injected into the lateral preoptic area increases IP with a latency for the response of approximately 10 min and achieves a peak response approximately 25 min after injection without inducing any changes in RC and arterial pressure (Lamy et al., 2021). Hence, the response observed with Ang-(1–7) infused intravenously in the current study could be dependent on direct action on the UB and/or be due to an action at the intrarenal level and/or through activation of Mas receptors in the lateral preoptic area.

In the present study, we could not test if the Ang-(1–7) administered intravenously was binding to Mas receptors in the UB by pharmacological blockade of these receptors. Preliminary findings in our laboratory (data not shown) indicated that the intravenous administration of A-779, a Mas receptors antagonist, even in the highest dose, causes the blockade of these receptors for less than 30 min. Even with an experimental protocol in which the administration of A-779 was followed by immediate administration of Ang-(1–7) intravenously, we would not be able to see if the IP response evoked by Ang-(1–7) could be attenuated as the blockade of Mas receptors had already quited. Thereafter, performing this experimental protocol has become unfeasible, which is a limitation of this study.

Micturition and urine storage depend on the coordination between the bladder and the urethra (de Groat, 1998; Andersson and Hedlund, 2002). An increasing number of people worldwide suffer from UB dysfunctions (Bettez et al., 2012; Dorsher and McIntosh, 2012; Ruffion et al., 2013). Nevertheless, the pharmacological approaches and therapies currently applied still cause many side effects. As we have shown that Ang-(1–7) acts in the UB increasing the IP, the Mas receptors and ACE-2 in the bladder could be considered to be novel possible targets for drug development and possible therapy in patients who suffer from urinary disorders.

In conclusion, our findings suggest that intravenous Ang-(1–7) can act in the UB, leading to an increase in the IP. This effect is similar to that observed by *in situ* administration onto the UB but with different latencies for the onset of the responses. In addition, the existence of ACE-2 in the UB suggests that Ang- (1–7) can be locally produced and is likely able to exert a paracrine or autocrine action in the UB binding to Mas receptors present in this organ.

## Data Availability

The original contributions presented in the study are included in the article/supplementary material, further inquiries can be directed to the corresponding author.
